# The effect of liquid type on the detection of airway invasion during swallow evaluation

**DOI:** 10.1007/s00405-025-10005-x

**Published:** 2026-01-21

**Authors:** Eiman Abu Bandora, Liav Hayat, Yarden Ashkenazi, Yael Oestreicher-Kedem, Nogah Nativ-Zeltzer, Yuval Nachalon

**Affiliations:** 1 Department of Otolaryngology, Head and Neck and Maxillofacial Surgery, Tel-Aviv Sourasky Medical Center, Tel Aviv, Israel; 2 Gray Faculty of Medical & Health Sciences, Tel Aviv University, Tel Aviv, Israel

## Abstract

**Objectives:**

To explore FEES findings obtained using different liquid types, water, cow’s milk, and soy milk, with respect to observed penetration, aspiration, and pharyngeal residue, and to describe agreement across liquids.

**Methods:**

Adult patients referred to a university-affiliated dysphagia clinic underwent standardized FEES using water, cow’s milk, and soy milk during single and consecutive swallows. Evaluations were performed jointly by a laryngologist and a speech-language pathologist (SLP). Outcome measures included the Penetration–Aspiration Scale (PAS) and the Yale Pharyngeal Residue Severity Rating Scale. Inter-rater reliability was assessed through blinded reassessment of 25% of examinations by an additional laryngologist and two SLPs.

**Results:**

Most swallows across all liquids were classified as safe. Unsafe PAS scores occurred numerically more frequently with cow’s milk (18.2–25.6%), than with water (6.9–11.6%) or soy milk (9.4–11.6%). For single swallows, a nominal overall difference across liquids was observed (Cochran’s Q = 6.00, p = 0.050), and for consecutive swallows an overall difference was also detected (Cochran’s Q = 8.00, p = 0.018). Pairwise comparisons were not statistically significant after correction for multiple testing. No statistically significant differences were identified between liquids for vallecular or pyriform sinus residue. Agreement in PAS scores across liquids ranged from low to moderate (κ = 0.291–0.609), and agreement for residue scores ranged from moderate to almost perfect (κ = 0.528–0.824). Inter-rater reliability was excellent (ICC = 0.93, 95% CI [0.90-0.95], p < 0.001).

**Conclusion:**

In this exploratory within-subject study, no statistically significant differences were observed in FEES penetration, aspiration, or pharyngeal residue scores when using water, cow’s milk, or soy milk. These findings provide preliminary descriptive data regarding the behavior of a commonly consumed plant-based liquid during FEES and may inform future research on liquid properties in swallowing assessment.

**Supplementary information:**

The online version contains supplementary material available at 10.1007/s00405-025-10005-x.

## Introduction

Flexible endoscopic evaluation of swallowing (FEES) is a key diagnostic tool for assessing oropharyngeal dysphagia, enabling detection of penetration, aspiration, and pharyngeal residue [1–6].The choice of liquid medium during FEES may influence detection of penetration, aspiration, and residue, as factors such as opacity, coating properties, and viscosity affect visualization [7, 8].Several studies have explored the impact of liquid color and opacity, comparing barium, various food dyes, and milk, yet findings regarding their effect on aspiration detection have been inconsistent [8, 10]. Opacity has also been mentioned previously as a factor impacting the visualization of medium during FEES, as non-translucent, opaque boluses are thought to be more easily seen during FEES when compared to non-opaque, clear boluses [11].

Butler et al. has demonstrated that thin liquids proprieties, including type, delivery methods, and bolus volume, significantly affect swallowing safety during FEES. In their work, PAS scores varied by liquid type and bolus size, with 2% and whole milk consistently producing higher PAS scores than skim milk or water, indicating that milk tends to reveal penetration and aspiration more frequently than water [12–14]. Importantly, higher PAS scores in this context reflect increased detection of penetration and aspiration events, a desirable characteristic for a medium used in diagnostic swallow evaluation. Another proposed property to affect FEES visualization is the coating effect, as it has been hypothesized to mark the path of bolus flow. Although not directly tested, this effect has been proposed with barium and white dyed water [10, 15–18]. However, the relative contribution of coating, opacity, and viscosity to airway invasion detection remains incompletely understood, and no single liquid property has been shown to consistently optimize FEES sensitivity across patient populations.

While dairy milk has been widely studied and used as opaque medium in FEES, its use is not always feasible given the increasing prevalence of veganism and lactose intolerance. To date, the potential utility of plant-based liquids such as soy milk, almond milk, or oat milk for FEES assessments has not been systematically investigated. Understanding whether these alternatives demonstrate similar detection patterns to cow milk could help clinicians tailor evaluations to patients’ dietary needs.

Soy milk was selected for this study due to its widespread availability and physical properties. Unlike almond or oat milk, commercially available soy milk has a protein and fat profile, as well as a total solid content, that more closely resemble cow’s milk, making it a pragmatic candidate for exploratory comparison [19, 20]. In terms of color, soy milk exhibits the closest whiteness index to cow’s milk, a characteristic determined by particle size, concentration, and chromophore levels. Importantly, with respect to viscosity, although values may vary depending on formulation and raw materials, soy milk typically demonstrates a viscosity which closely matches that of cow’s milk [20]. These characteristics suggested that soy milk may provide adequate endoscopic visualization during FEES. Additionally, the similar cost and accessibility of soy milk relative to regular milk further supported its selection for this study.

The present study aimed to explore how soy milk performs relative to water and cow’s milk during FEES with respect to observed penetration, aspiration, and pharyngeal residue within a within-subject design, with water serving as a reference.

## Methods

After obtaining institutional review board approval (0583-22-TLV), forty-four consecutive adult patients referred for swallow evaluation at a dysphagia clinic were assessed by an experienced speech-language pathologists (SLPs) and laryngologists. Detailed demographic and clinical information were collected for each patient. For each enrolled participant, all three liquid types were administered during the same FEES examination, allowing each patient to serve as their own control.

Sample size was determined based on power analysis, assuming α=0.05, power=0.80, and a medium expected effect size of 0.5 for detecting overall differences between liquid types, resulting in a minimum requirement of 42 participants.

Inclusion criteria were: [1] age ≥18 years [2], clinical indication for FEES evaluation, and [3] ability to complete the full protocol with all three liquid types. Exclusion criteria included: [1] severe agitation or inability to cooperate with examination [2], severe epistaxis, and [3] known allergy to any test substances.

### Fiberoptic endoscopic evaluation of swallowing (FEES) assessment

FEES was performed using a video laryngoscope (VNL11-J10, PENTAX Europe GmbH, Hamburg, Germany), with recordings captured on a workstation (Orpheus-Medical system Software Version 11.0.70.2). The laryngoscope was inserted transnasally without the application of a topical anesthetic or vasoconstrictor, and was positioned either at the junction of the nasopharynx and oropharynx or within the oropharynx according to anatomical constraints, maintaining patient comfort and examiner judgment. For each patient, endoscope position was kept consistent across all liquid trials.

Participants then completed standardized swallowing tasks and were given three test liquids: colored water (Magic Colors Pro Food Coloring, Magic Colors Ltd., Beit Shemesh, Israel), soy milk (Alpro 2%, Alpro S.A., Ghent, Belgium), and cow’s milk (Tnuva 3%, Tnuva Ltd., Petah Tikva, Israel). All liquids corresponded to International Dysphagia Diet Standardization Initiative (IDDSI) Level 0. Each liquid was administered in two conditions, single sips of 10 ml, followed by consecutive swallows of 30 ml, during which patients were instructed to swallow continuously without stopping. Between different liquid types, patients were instructed to perform several swallows to facilitate bolus clearance and minimize carryover of residue from prior trials.

After each swallow, the pharynx was examined for residue in the vallecula and pyriform sinus, graded using the Yale Pharyngeal Residue Severity Rating Scale [21]. Aspiration and penetration were assessed and graded using the Penetration-Aspiration Scale (PAS) [22]. Primary scoring of PAS and residue was performed in real time during the FEES examination by an experienced laryngologist and SLP, reflecting routine clinical practice.

To assess inter-rater reliability, 25% of examinations were randomly selected, de-identified, segmented by swallowing task, and randomized. These were independently re-evaluated offline by two additional SLPs and an experienced laryngologist. Reviewers performing the offline reliability assessment were blinded to patient identity and clinical background, and video clips were presented in randomized order. Complete blinding to liquid identity was not feasible due to inherent visual differences between water and milk-based liquids.

### Statistical analysis

Given the within-subject design and ordinal nature of the rating scales, all analyses were performed using paired, non-parametric statistical methods. Continuous demographic variables are presented as mean and standard deviation. Categorical variables are presented as frequencies and percentages. PAS and Yale Pharyngeal Residue Severity Rating Scale scores were analyzed as ordinal categorical data rather than as continuous values, in line with the design of the scale. For descriptive purposes, PAS outcomes were grouped into three categories: safe (PAS 1–2), penetration (PAS 3–5), and aspiration (PAS 6–8). To facilitate comparison, analyses were performed both with three categories and in a dichotomized fashion (safe [PAS 1–2] vs. abnormal [PAS 3–8]). Vallecular and pyriform sinus residue were assessed using the Yale Pharyngeal Residue Severity Rating Scale, scored from 1 (none) to 5 (severe). For statistical analysis, scores were summarized descriptively across all categories and dichotomized into none/trace (scores 1–2) versus mild–severe residue (scores 3–5). For dichotomized PAS outcomes (“safe” = PAS 1- 2; “unsafe” = PAS ≥3), comparisons across liquids were evaluated using Cochran’s Q test, followed by McNemar’s tests with Bonferroni adjustment for post-hoc pairwise contrasts.

To compare swallowing outcomes across the three liquid types (water, cow’s milk, soy milk), Friedman tests were applied to PAS and Yale residue scores for both single and consecutive swallows. When the Friedman test reached statistical significance, pairwise Wilcoxon signed-rank tests with Bonferroni correction were conducted to identify specific between-liquid differences.

Agreement between liquids was assessed using quadratically weighted kappa (κw) for each pairwise comparison (water vs. cow’s milk, water vs. soy milk, cow’s milk vs. soy milk). Agreement was evaluated separately for PAS scores (single and consecutive swallows) and Yale vallecular and pyriform residue ratings. Interpretation of κ values followed the Landis and Koch criteria: slight (0.00 - 0.20), fair (0.21 - 0.40), moderate (0.41 - 0.60), substantial (0.61 - 0.80), and almost perfect (>0.80) agreement. Inter-rater reliability was evaluated using intra-class correlation (ICC). Statistical significance was set at *p*<0.05 for all analyses. All analyses were performed using SPSS software (Version 25, IBM Corp., Armonk, NY).

## Results

**Fig. 1:**
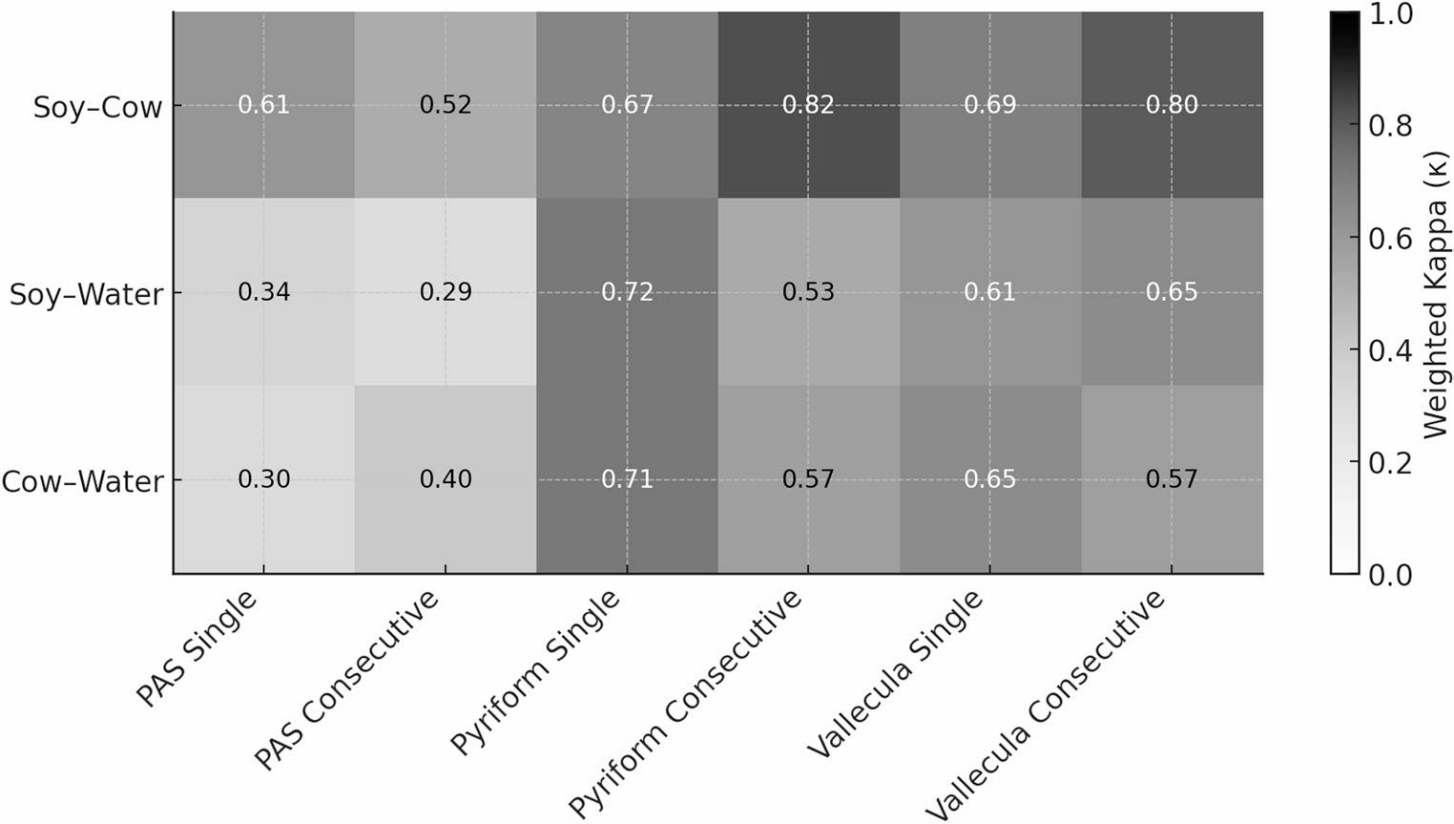
Heat map of quadratically weighted kappa values quantifying agreement between liquid pairs (water, cow’s milk, soy milk) for PAS scores (single and consecutive swallows) and Yale pyriform and vallecular residue ratings. Darker shading indicates higher agreement

A total of 44 patients underwent FEES evaluation, of whom 63.63% were males, with a mean age of 61.68 ± 13.09 years. Patients’ characteristics and dysphagia etiology are summarized in Table 1. In total, 264 swallows were analyzed. PAS and Yale residue scores for each liquid type are presented in Fig. 1.

**Table 1:** Patient characteristics

Characteristic	
Age, years (mean ±SD)	61.68 ± 13.92
Gender, N (%)	
Male	28 (63.63)
Female	16 (36.36)
Related Medical Conditions/Referral Symptoms, N (%)	
Idiopathic Dysphagia	8 (18.18)
Parkinson’s Disease	7 (15.9)
Chronic Cough	6 (13.63)
s/p Head and Neck Cancer*	5 (11.36)
Intracranial Pathologies	4 (9.09)
Neuromuscular Pathologies	3 (6.81)
Dementia	2 (4.54)
Cerebrovascular Accident (CVA)	2 (4.54)
Direct Laryngoscopy/Bronchoscopy Findings	2 (4.54)
Globus Pharyngeus	2 (4.54)
Epilepsy	1 (2.27)
EGJ Outflow Narrowing**	1 (2.27)
Presbylarynx	1 (2.27)

*s/p: status post ** *EGJ:* Esophagogastric junction

### Prevalence of abnormal PAS score by liquid type and swallow type

The majority of swallows were classified as safe (PAS 1–2) across all liquid types. For single swallows, water was associated with safe PAS scores in 41 patients (93.1%) while 3 patients (6.9%) demonstrated unsafe PAS scores (PAS ≥ 3). Cow’s milk was associated with safe swallows in 36 patients (81.8%), with 8 patients (18.2%) demonstrating unsafe PAS scores. Soy milk was associated with safe swallows in 39 patients (88.6%), with 4 patients (9.1%) demonstrating unsafe PAS scores. (Table 2)

**Table 2:** Prevalence of unsafe PAS score across liquid types and swallow conditions

	Single Swallow		Consecutive Swallow	
*N* (%)	PAS (1–2)	PAS (3–8)	PAS (1–2)	PAS (3–8)
Water	41(93.1)	3(6.9)	38(88.4)	5(11.6)
Cow milk	36(81.8)	8(18.2)	32(74.4)	11(25.6)
Soy milk	39(90.6)	4(9.4)	38(88.4)	5(11.6)

For consecutive swallows, water demonstrated safe PAS scores in 38 patients (86.4%), with unsafe PAS scores observed in 5 patients (11.6%), Cow’s milk demonstrated safe PAS scores in 32 patients (72.7%), with unsafe PAS scores observed in 11 patients (25.6%). Soy milk demonstrated safe PAS scores in 38 patients (86.4%), with unsafe PAS scores observed in 5 patients (11.6%).

For both single and consecutive swallows, the proportion of unsafe PAS scores was numerically higher with cow’s milk compared with water and soy milk. For single swallows, comparison of dichotomized PAS outcomes across liquids showed an overall difference that did not remain significant after correction (Cochran’s Q = 6.00, *p* = 0.050). None of the pairwise McNemar comparisons between water, cow’s milk, and soy milk reached statistical significance after Bonferroni correction (*p* = 0.125, *p* = 1.00, and *p* = 0.125).

For consecutive swallows, Cochran’s Q indicated an overall difference in the prevalence of unsafe PAS scores among liquids (Q = 8.00, *p* = 0.018). Pairwise comparisons demonstrated a higher proportion of unsafe PAS scores with cow’s milk compared with soy milk (uncorrected *p* = 0.031), though this difference did not remain statistically significant after Bonferroni correction. No statistically significant pairwise differences were observed between cow’s milk and water or between soy milk and water.

### Yale pyriform and vallecula scores stratified by medium and swallow type

Pharyngeal residue was uncommon across all liquids. For single swallows, vallecular residue was absent or trace (scores 1–2) in all patients for water (100%), in 42 patients (95%) for cow’s milk, and in all patients for soy milk (100%). Mild–severe vallecular residue (scores 3–5) was observed in one patient (5%) with cow’s milk and was not observed with water or soy milk. For consecutive swallows, vallecular residue remained low across liquids, with absent or trace residue observed in 42 patients (95%) for water, 41 patients (95%) for cow’s milk, and 41 patients (95%) for soy milk. (Table 3 A)

**Table 3:** Yale residue score with different liquid types and swallow conditions

A. Vallecular Residue				
	Single swallow		Consecutive swallow	
N (%)	None/trace (1–2)	Residue (3–5)	None/trace (1–2)	Residue (3–5)
Water	44(100)	0(0)	42(97.6)	1(2.4)
Cow milk	42(97.7)	1(2.3)	41(95.3)	2(4.7)
Soy milk	43(100)	0(0)	41(95.3)	2(4.7)
B. Pyriform Sinus Residue				
	**Single swallow**		**Consecutive swallow**	
	**None/trace (1–2)**	**Residue (3–5)**	**None/trace (1–2)**	**Residue (3–5)**
Water	41(93.1)	3(6.9)	38(88.3)	5(11.7)
Cow milk	42(97.7)	1(2.3)	39(90.7)	4(9.3)
Soy milk	42(97.7)	1(2.3)	40(93)	3(7)

Pyriform sinus residue followed a similar pattern. For single swallows, absent or trace residue was observed in 41 patients (93%) for water, 42 patients (95%) for cow’s milk, and 42 patients (95%) for soy milk. For consecutive swallows, absent or trace residue was present in 38 patients (87%) for water, 39 patients (91%) for cow’s milk, and 40 patients (93%) for soy milk. (Table 3B)

No statistically significant differences were identified across liquids for vallecular residue (single swallows: Cochran’s Q = 2.00, *p* = 0.368, consecutive swallows: Cochran’s Q = 0.50, *p* = 0.779) or for pyriform sinus residue (single swallows: Cochran’s Q = 1.00, *p* = 0.607, consecutive swallows: Cochran’s Q = 0.50, *p* = 0.78).

### Agreement between liquid types

Agreement between PAS and Yale residue scores obtained using different liquid types was evaluated using quadratically weighted kappa (κw). For PAS scores, κw values indicated low to moderate agreement across liquid pairs during both single swallows (κw = 0.305–0.609) and consecutive swallows (κw = 0.291–0.520). In contrast, agreement for Yale residue ratings was consistently higher. κw values for pyriform sinus residue ranged from 0.673 to 0.715 during single swallows and from 0.528 to 0.824 during consecutive swallows. Vallecular residue ratings showed κw values of 0.609–0.690 for single swallows and 0.573–0.797 for consecutive swallows. (Table 4; Fig. 1)

**Table 4:** Agreement between liquid types using quadratically weighted kappa (κw)

Measurement	Liquid Comparison	κ	Level of Agreement
PAS – Single Swallow	Soy milk and cow milk	0.609	Moderate-Substantial
	Soy milk and water	0.343	Fair
	Cow milk and water	0.305	Fair
PAS – Consecutive Swallow	Soy milk and cow milk	0.520	Moderate
	Soy milk and water	0.291	Fair
	Cow milk and water	0.404	Fair-Moderate
Yale Pyriform – Single	Soy milk and cow milk	0.673	Substantial
	Soy milk and water	0.717	Substantial
	Cow milk and water	0.715	Substantial
Yale Pyriform – Consecutive	Soy milk and cow milk	0.824	Almost Perfect
	Soy milk and water	0.528	Moderate
	Cow milk and water	0.570	Moderate
Yale Vallecula – Single	Soy milk and cow milk	0.690	Substantial
	Soy milk and water	0.609	Moderate-Substantial
	Cow milk and water	0.652	Substantial
Yale Vallecula – Consecutive	Soy milk and cow milk	0.797	Substantial-Almost Perfect
	Soy milk and water	0.649	Substantial
	Cow milk and water	0.573	Moderate-Substantial

Analysis of ordinal PAS scores using Friedman tests demonstrated no statistically significant differences across liquids for single swallows (χ² = 7.14, *p* = 0.08) or consecutive swallows (χ² = 2.45, *p* = 0.29). Post-hoc Wilcoxon signed-rank tests with Bonferroni correction did not identify significant pairwise differences between liquid types.

Similarly, no statistically significant differences were observed across liquids for ordinal Yale vallecular or pyriform sinus residue scores for either single or consecutive swallows.(Supplementary Table S1).

### Inter-rater reliability

The inter-rater reliability for PAS and Yale residue scoring was excellent, with an ICC of 0.93 (95% CI [0.90–0.95.90.95], *p*<0.001).

## Discussion

Since its introduction in 1988, FEES has become a widely used tool for evaluating pharyngeal dysphagia, offering direct visualization of swallowing mechanics [15, 16]. While advances in optical and digital imaging have enhanced aspiration detection, the choice of liquid medium remains a key variable influencing detection of airway invasion [7]. Only a limited number of studies have explored how liquid properties, such as color, opacity, viscosity, volume, coating, and delivery method, affect penetration and aspiration detection during FEES [7–9, 13, 23]. Nevertheless, no universally accepted standard exists for the standardized test medium [12].

Against this background, our study evaluated FEES findings obtained using water, soy milk, and cow’s milk with respect to observed penetration, aspiration, and pharyngeal residue within a within-subject design. Given the growing number of individuals with dietary restrictions, such as veganism and lactose intolerant, it is essential to explore how plant-based liquids such as soy milk behave during FEES compared with commonly used thin liquids. To our knowledge, this is the first within-subject study to directly compare these liquids, thereby adding to the growing body of evidence on how liquid properties affect FEES-based swallowing assessments.

### Opacity and visualization in FEES

Opacity has been proposed as a key determinant of aspiration detection, as non-translucent liquids may enhance endoscopic visualization [11]. Butler and colleagues consistently reported higher PAS scores for milk compared to water, supporting the role of opacity in aspiration detection [12, 13, 23] For example, in one study of 14 healthy older adults completing 448 swallows across four liquid types (water, skim, 2%, and whole milk), PAS scores were significantly higher with 2% and whole milk than with skim milk and water [13], a property considered advantageous for visualization during diagnostic swallow assessment. Similarly, in a larger cohort of 203 adults, PAS scores were significantly higher for milk compared with water, with effects most pronounced in participants over 70 years old, whereas younger adults showed minimal differences [23].

In our cohort, cow’s milk showed a trend toward higher PAS scores than water and soy milk, consistent with earlier findings [13, 23], but these differences did not remain statistically significant after pairwise correction. This may reflect either limited statistical power or true similarity in observed findings, given that soy milk and cow’s milk share similar opacity and coating properties.

### Coating effects and bolus tracking

The coating properties of a liquid may influence bolus visibility and residue assessment. Pisegna et al. highlighted that standardized residue rating methods improve detection reliability regardless of the test medium [17]. Kelly et al. found that residue perception varied depending on liquid properties, underscoring the role of visualization on assessment [16]. Curtis et al. demonstrated that liquids with coating effects, such as white-dyed water, improved detection of pharyngeal residue and unsafe swallows compared to liquids without coating effects, including milk and blue-dyed water [7]. They concluded that coating appears to be the most important factor for maximizing sensitivity of detecting pharyngeal residue and airway invasion during FEES.

In the present study, no statistically significant differences were observed in vallecular or pyriform sinus residue scores across cow’s milk, soy milk, and water. Although numeric differences in PAS distributions were observed across liquid types, these did not remain statistically significant after correction for multiple comparisons. Accordingly, the present findings do not demonstrate differences in airway invasion or residue outcomes between liquids.

### Liquid composition and PAS scores

Previous research suggests that liquid composition also influences swallowing physiology, with higher lipid content and viscosity leading to greater penetration and aspiration. Butler et al. (2009) first documented that milk boluses produced different swallowing patterns compared to water in 44 healthy adults, with penetration and aspiration events more frequent in older participants. In a subsequent study, Butler and colleagues compared water (approximately 1.02 × 10^−3^ Pa•s), skim milk (approximately 1.18 × 10^−3^ Pa•s), 2% milk (approximately 1.22 × 10^−3^ Pa•s), and whole milk (approximately 1.26 × 10^−3^ Pa•s). They found that 2% and whole milk consistently yielded higher PAS scores than both water and skim milk, likely due to increased viscosity and lipid content. The researchers proposed two potential mechanisms: improved visualization due to greater milk’s opacity and differences in swallowing dynamics related to viscosity and lipid composition [13]. These findings were reinforced in their 2018 study of 203 healthy adults, which confirmed that 2% and whole milk produced significantly higher PAS scores compared to water and skim milk, with effects most evident in adults over 70 years old.

In the present study we did not identify significant PAS differences between water, soy milk, and cow’s milk, despite the differences in nutritional composition mainly between water and milk/soy milk. (19)(20) A possible explanation would be that swallowing safety is influenced by multiple interacting factors—including protein composition, viscosity, bolus texture, and even modern imaging quality—rather than any single compositional feature. Thus, the diagnostic performance of liquids in FEES may depend more on rheology and visualization characteristics than on specific nutritional properties.

Taken together, these results highlight the complexity of liquid properties in swallowing assessment, and suggest that swallowing safety is shaped by an interplay of fat, protein, viscosity, and opacity, rather than any single compositional variable [23]. Further research is warranted to explore these mechanisms, particularly the role of plant-based proteins in swallowing physiology.

This study has several limitations that should be considered when interpreting the findings. First, although sample size met the requirements of the power analysisthe number of penetration and aspiration events observed was small, limiting statistical power for detecting between-liquid differences in airway invasion. Second, all test liquids were administered as thin liquids (IDDSI level 0), which may inherently restrict the ability to detect differences in residue or airway invasion within this classification. Third, primary scoring of penetration, aspiration, and residue was performed in real time during FEES, reflecting routine clinical practice, but introducing potential expectation and rater bias. Although a subset of recordings underwent blinded offline re-evaluation to assess inter-rater reliability, complete blinding to liquid identity was not feasible due to inherent visual differences between liquids. Fourth, endoscope positioning varied between the nasopharynx and oropharynx according to patient anatomy and examiner judgment. Although position was kept consistent within each patient, nasopharyngeal positioning may intermittently obscure visualization during swallowing due to palatal elevation. Additionally, FEES is subject to brief loss of visualization during the whiteout phase, which may lead to missed aspiration events, particularly for rapidly flowing thin liquids. The study did not include a second instrumental assessment, such as videofluoroscopic swallowing study, for cross-validation of aspiration events. Finally, liquids were administered sequentially rather than in a randomized order, raising the possibility of carryover or fatigue effects despite the use of neutral swallows between trials. The use of single brands of cow’s milk and soy milk and the single-center design may further limit generalizability.

Future studies should incorporate randomized liquid sequences, longer washout periods, multiple brands of plant-based liquids, and dual-modality instrumental assessment to enhance generalizability and further validate our findings.

## Conclusions

In this exploratory within-subject study, FEES findings obtained using water, cow’s milk, and soy milk did not differ significantly with respect to penetration, aspiration, or pharyngeal residue scores. Within the study cohort and protocol employed, soy milk demonstrated FEES findings that did not differ statistically from those observed with commonly used thin liquids. These results provide preliminary descriptive data on the behavior of a plant-based liquid during FEES. Further studies with larger cohort are warranted to fully examine the results of these studies and verify its suggestive results.

## Supplementary information

Below is the link to the electronic supplementary material.

Supplementary File 1(DOCX 18.6 KB)

## Data Availability

The datasets analyzed during the current study involve patient clinical and instrumental swallowing data and are therefore not publicly available due to institutional and privacy restrictions. De-identified data may be made available from the corresponding author upon reasonable request.

## References

[1] Labeit B, Ahring S, Boehmer M et al (2022) Comparison of simultaneous swallowing endoscopy and videofluoroscopy in neurogenic dysphagia. J Am Med Dir Assoc 19:1360–1366. 10.1016/j.jamda.2022.05.019 10.1016/j.jamda.2021.09.026 34678269

[2] Rao N, Brady SL, Chaudhuri G, Donzelli JJ (2003) Gold-standard? Analysis of the videofluoroscopic and fiberoptic endoscopic swallow examinations. J Appl Res Clin Exp Ther 3(1):1–6

[3] Colodny N (2002) Interjudge and intrajudge reliabilities in fiberoptic endoscopic evaluation of swallowing (FEES®) using the penetration-aspiration scale: a replication study. Dysphagia 17:88–100. 10.1007/s00455-001-0113-1 10.1007/s00455-002-0073-4 12355146

[4] Kollmeier BR, Keenaghan M (2017) Aspiration risk. Med Clin North Am 101:525–535. 10.1016/j.mcna.2016.12.006

[5] Marik PE, Kaplan D (2003) Aspiration pneumonia and dysphagia in the elderly. Chest 124(1):328–336. 10.1378/chest.124.1.328 12853541 10.1378/chest.124.1.328

[6] Rofes L, Arreola V, Almirall J et al (2011) Diagnosis and management of oropharyngeal dysphagia and its nutritional and respiratory complications in the elderly. Gastroenterol Res Pract 2011:818979. 10.1155/2011/818979 20811545 10.1155/2011/818979 PMC2929516

[7] Curtis JA, Seikaly ZN, Dakin AE, Troche MS (2021) Detection of aspiration, penetration, and pharyngeal residue during FEES: comparing the effects of color, coating, and opacity. Dysphagia 36:207–215. 10.1007/s00455-020-10133-4 32394024 10.1007/s00455-020-10131-0

[8] Marvin S, Gustafson S, Thibeault S (2016) Detecting aspiration and penetration using FEES with and without food dye. Dysphagia 31:498–504. 10.1007/s00455-016-9703-0 26993648 10.1007/s00455-016-9703-0

[9] Leder SB, Acton LM, Lisitano HL, Murray JT (2005) Fiberoptic endoscopic evaluation of swallowing (FEES) with and without blue-dyed food. Dysphagia 20:157–162. 10.1007/s00455-005-0019-4 16172826 10.1007/s00455-005-0009-x

[10] Curtis J, Perry S, Troche MS (2019) Detection of airway invasion during flexible endoscopic evaluations of swallowing: comparing barium, blue dye, and green dye. Am J Speech Lang Pathol 28:1262–1268. 10.1044/2019_AJSLP-19-0005 10.1044/2018_AJSLP-18-0119 31136233

[11] Leder SB, Murray JT (2008) Fiberoptic endoscopic evaluation of swallowing. Phys Med Rehabil Clin N Am 19:787–801. 10.1016/j.pmr.2008.05.003 18940641 10.1016/j.pmr.2008.05.003

[12] Butler SG, Stuart A, Kemp S (2009) Flexible endoscopic evaluation of swallowing in healthy young and older adults. Ann Otol Rhinol Laryngol 118(2):99–106. 10.1177/000348940911800204 19326759 10.1177/000348940911800204

[13] Butler SG, Stuart A, Case LD, Rees C, Vitolins M, Kritchevsky SB (2011) Effects of liquid type, delivery method, and bolus volume on penetration-aspiration scores in healthy older adults during FEES. Ann Otol Rhinol Laryngol 120(1):1–7. 10.1177/000348941112000101 21675583 10.1177/000348941112000502

[14] Butler SG, Stuart A, Leng X, Rees C, Williamson J, Kritchevsky SB (2010) Factors influencing aspiration during swallowing in healthy older adults. Laryngoscope 120:2147–2152. 10.1002/lary.21116 20938951 10.1002/lary.21116 PMC3780773

[15] Langmore SE (2017) History of fiberoptic endoscopic evaluation of swallowing for evaluation and management of pharyngeal dysphagia: changes over the years. Dysphagia 32:27–38. 10.1007/s00455-016-9775-x 28101663 10.1007/s00455-016-9775-x

[16] Kelly AM, Leslie P, Beale T, Payten C, Drinnan MJ (2006) Fibreoptic endoscopic evaluation of swallowing and videofluoroscopy: does examination type influence perception of pharyngeal residue severity? Clin Otolaryngol 31(5):425–432. 10.1111/j.1749-4486.2006.01285.x 17014453 10.1111/j.1749-4486.2006.01292.x

[17] Pisegna JM, Kaneoka A, Coster WJ, Leonard R, Langmore SE (2020) Residue ratings on FEES: trends for clinical application of residue measurement. Dysphagia 35:164–172. 10.1007/s00455-019-10089-8 10.1007/s00455-019-10089-8 31912241

[18] Kaneoka AS, Langmore SE, Krisciunas GP et al (2013) The Boston Residue and Clearance Scale: preliminary reliability and validity testing. Folia Phoniatr Logop 65:312–317. 10.1159/000365006 25033761 10.1159/000365006

[19] Collard KM, McCormick DP (2021) A nutritional comparison of cow’s milk and alternative milk products. Acad Pediatr 21(6):1067–1069. 10.1016/j.acap.2020.12.007 33373745 10.1016/j.acap.2020.12.007

[20] Reyes-Jurado F, Soto-Reyes N, Dávila-Rodríguez M et al (2021) Plant-based milk alternatives: types, processes, benefits, and characteristics. Food Rev Int 39(4):2320–2351. 10.1080/87559129.2021.1952421

[21] Neubauer PD, Rademaker AW, Leder SB (2015) The Yale Pharyngeal Residue Severity Rating Scale: an anatomically defined and image-based tool. Dysphagia 30:521–528. 10.1007/s00455-015-9631-4 26050238 10.1007/s00455-015-9631-4

[22] Rosenbek JC, Robbins J, Roecker EB, Coyle JL, Wood JL (1996) A penetration–aspiration scale. Dysphagia 11:93–98. 10.1007/BF00417897 8721066 10.1007/BF00417897

[23] Butler SG, Stuart A, Markley L, Feng X, Kritchevsky SB (2018) Aspiration as a function of age, sex, liquid type, bolus volume, and bolus delivery across the healthy adult life span. Ann Otol Rhinol Laryngol 127(1):21–32. 10.1177/0003489417747084 29188729 10.1177/0003489417742161

