# Supplementary material for: The effect of liquid type on the detection of airway invasion during swallow evaluation
Source: Eur Arch Otorhinolaryngol. 2026 Jan 21;283(3):1803–10. doi: 10.1007/s00405-025-10005-x (PMC13002630; doi:10.1007/s00405-025-10005-x)
Supplement: Supplementary file 1 — (DOCX 18.6 KB) [file 405_2025_10005_MOESM1_ESM.docx]

Supplementary:

**Table S1. Statistical comparison of liquid types across PAS and Yale residue scores**

| **Outcome Measure** | **Swallow Type** | **Friedman χ²** | **p-value** | **Post-hoc Wilcoxon Comparisons (Z, p)** | **Bonferroni-correction** |
| --- | --- | --- | --- | --- | --- |
| **PAS** | **Single** | 7.143 | 0.028 | Water vs Milk: Z = −2.084, p = .037 | **No** (α = .017) |
|  |  |  |  | Water vs Soy: Z = −1.35, p = .176 | No |
|  |  |  |  | Milk vs Soy: Z = −1.518, p = .129 | No |
| **PAS** | **Consecutive** | 2.45 | 0.29 | Post-hoc not required | – |
| **Vallecular residue (Yale)** | **Single** | 0.56 | 0.756 | Not performed (ns) | – |
| **Vallecular residue (Yale)** | **Consecutive** | 3.24 | 0.198 | Not performed (ns) | – |
| **Pyriform residue (Yale)** | **Single** | 2 | 0.368 | Not performed (ns) | – |
| **Pyriform residue (Yale)** | **Consecutive** | 1.76 | 0.41 | Not performed (ns) | – |

Friedman tests were performed on ordinal PAS scores. Cochran’s Q tests reported in the main text reflect dichotomized PAS outcomes.
